# Book Review: Cultivating Teacher Resilience: International Approaches, Applications, and Impact

**DOI:** 10.3389/fpsyg.2021.706751

**Published:** 2021-07-27

**Authors:** Rui Xu

**Affiliations:** School of College English Teaching and Research, Henan University, Kaifeng, China

**Keywords:** positive psychology, teacher-student interpersonal variables, stress-free environment, teacher resilience, people-centered dispositions

Strongly rooted in the theoretical underpinnings and pedagogical implications of positive psychology, *teacher resilience* is one of the quintessential constituents of teacher-student interpersonal variables that can substantially contribute to the learners' success, motivation, and achievement. It has also been postulated that teacher effectiveness, teachers' classroom practices, and teacher-student relationships in a stress-free environment can have a tremendous impact on students' learning and engagement. Although there has been a growing body of literature on teacher resilience, “rich narratives and systematic knowledge that explicate the important role that teacher education plays in promoting resilience in early career teachers remain, surprisingly, scarce.” (p. vii). Additionally, teacher resilience is significant in that teachers need to tackle such problems as shortage, turnover, and attrition which can have deleterious effects on students' engagement, motivation, and success. Reconciling theoretical conceptualizations and pedagogical implications of teacher resilience in thorough ways and elucidating how multifarious disciplinary approaches and educational milieus conceptualize teacher resilience are the foci of *Cultivating Teacher Resilience: International Approaches, Applications and Impact*, edited adeptly by Caroline F. Mansfield. Being inspired by two Australian projects: *Building Resilience in Teacher Education (BRiTE)* (Mansfield et al., 2016) and *Staying BRiTE: Promoting Resilience in Higher Education* (Mansfield, 2016), this research-informed and evidence-based compendium is definitely timely and opportune.

The book is organized into three parts, encompassing 18 chapters, written by a gamut of distinguished researchers. Part I, *Foundations*, including three chapters, lays the foundations of these two projects by presenting a comprehensive overview of the volume (Chapter 1), illuminating and appraising the different ways resilience has been conceptualized, pinpointing the merits afforded by multidimensional perspectives (Chapter 2), as well as contextualizing and exemplifying the development of BRiTE and Staying BRiTE modules (Chapter 3) to boost “pre-service teachers' development of resilience-related skills and strategies, through experiential and online learning” (p. 3).

Part II, *Implementation and Applications*, comprising eight chapters, focuses on implementing particular aspects of cultivating teacher resilience in a range of contexts. Being enlightened with *Staying BRiTE*, Chapters 4 and 5, set in the Australian context, indicate how resilience learning was implemented in teacher education with pre-service teachers and post-graduate students, highlighting that people-centered dispositions and resilience are interwoven. Moving to an international scale, Chapter 6 investigates the outcomes of the BRiTE modules for beginning teachers' resilience in the USA, accentuating that the topics of the modules were fruitful for pre-service teachers. Reporting the results of a professional learning program on how to nurture and nourish resilience among teachers in the Portugal context is the focus of Chapter 7. Chapter 8 seems unique in that it incorporates the role of mentors in beginning teachers' resilience, foregrounding its positive influence on confidence, stress control, and commitment. Chapter 9, set in the Netherlands, presents the potential of the BRiTE modules and the rationale for its approach and implementation. Elaborating on the specific online mindfulness practices and exploring the relationship between mindfulness and resilience is the objective of Chapter 10. Embarking on the positive psychology lens, Chapter 11 closes this section by justifying how resilience and well-being are interrelated.

Part III, *Future Directions*, containing seven chapters, signposts the potential directions for future research. Drawing on the mixed-methods longitudinal mindfulness investigation among Australian school principals, Chapter 12 reports the impact of the program on their self-compassion, self-care, and greater resilience. Interestingly, Chapter 13 delineates that early-career casual teachers' development of teacher identity and their resilience are correlated. Informed by Job Demands-Resources (JD-R) theory, Chapter 14 provides new insights and avenues for future research by elucidating how teachers overcome adversity and demands at work. Innovatively, Chapter 15 intermingles BRiTE modules with Simlab™ (human in the loop synchronous simulation) using augmented reality experiences by inviting pre-service teachers to utilize the content from the BRiTE modules and rehearse their skills in a micro-teaching context with avatars. Proposing a conceptual framework for teacher resilience, Chapter 16 convincingly problematizes existing teacher resilience research because of overwhelming reliance on self-report measures, suggesting that future studies need to embark on more objective indicators of teacher resilience. Although the bulk of the previous chapters has concentrated on pre-service teachers, the penultimate chapter, capitalizing on the socio-ecological model, focuses on what sustains and challenges Australian teacher educators in their work. The closing chapter of this volume draws together fundamental themes and makes recommendations for future directions.

In many ways, Mansfield and all the venerable authors in this volume should be congenially praised for such a well-rounded collection of a conceptually robust and empirically rigorous range of national and international studies in contributing to two prominent projects in teacher education in Australia: *BRiTE* and *Staying BRiTE*. Firstly, being inspired by the systematic synthesis of the research insights, the chapters have employed a diverse range of methodologies, contexts, impacts, and applications to implement teacher resilience from different perspectives. Secondly, the poem at the beginning of each part is another engaging and lively creativity that visualizes different aspects of resilience and provides an intellectual reflection on the experience of resilience and growth. Thirdly, from the socio-ecological perspective and keeping in mind that “teaching is a culturally embedded conception and practice” (p. viii), the volume sagaciously encompassed studies from diverse contexts. However, had the book included cross-cultural studies on how teacher resilience is conceptualized and manifested, we would have gained more insights into the intricate role of contexts.

We confidently recommend this comprehensive volume to whoever is enthusiastic about upgrading standards and quality in teacher education. It is hoped that this volume makes a substantial contribution by enabling teachers to become world-class teachers through fostering and cultivating their resilience.

## Author Contributions

The author confirms being the sole contributor of this work and has approved it for publication.

## Conflict of Interest

The author declares that the research was conducted in the absence of any commercial or financial relationships that could be construed as a potential conflict of interest.

## Publisher's Note

All claims expressed in this article are solely those of the authors and do not necessarily represent those of their affiliated organizations, or those of the publisher, the editors and the reviewers. Any product that may be evaluated in this article, or claim that may be made by its manufacturer, is not guaranteed or endorsed by the publisher.
